# Social relationships and grit in English as a foreign language learning among high school students: A three-wave longitudinal study

**DOI:** 10.3389/fpsyg.2022.1038878

**Published:** 2022-10-03

**Authors:** Tianxue Cui, Yanchao Yang

**Affiliations:** ^1^Faculty of Education, University of Macau, Macau, Macau SAR, China; ^2^Qinggong College, North China University of Science and Technology, Tangshan, Hebei, China

**Keywords:** teacher-student relationship, peer relationship, grit, EFL learning, reciprocal relations

## Abstract

This study examined the longitudinal reciprocal relations between students’ grit and their perceptions of social relationships (teacher-student and peer relationships) in studying English as a foreign language (EFL). A total of 2,435 students from Grades 10-11 in China (M_age_ = 16.40 years old, 54.2% female) participated in the study on three occasions across 1 year. The three-wave cross-lagged analytic model results indicated that (a) peer relationship and grit reciprocally enhanced each other across both intervals; (b) the teacher-student relationship at Time 2 was influenced by Time 1 grit, but not vice versa. Nevertheless, the teacher-student relationship transactionally facilitated each other during the second interval (from Time 2 to Time 3). The multiple-group confirmatory factor analysis (MG-CFA) results indicated that such relations remained stable across gender. The study’s findings contribute to ongoing research delineating the dynamic system between social relationships and grit in EFL learning. It also reveals that males and females benefit similarly during social communications with peers and their English language teachers. Implications for educational practices were discussed.

## Introduction

As the world is experiencing hardship brought by the COVID-19 pandemic, positive psychological elements such as grit become essential for coping with adversities. The burgeoning of research on grit indicates a growing conviction that grit is a vital factor in dealing with adversities and achieving success for academic and non-academic issues (e.g., Von Culin et al., 2014; Blalock et al., 2015; Buckingham and Richardson, 2020). Grit, articulated by Duckworth and Quinn (2009), is defined as “trait-level perseverance and passion for long-term goals (p.166).” Grit includes two main definitional components: perseverance of effort and consistency of interest. Perseverance of effort refers to people’s continuing efforts in pursuing long-term goals, and consistency of interest refers to an individual’s ability to insist on the same goal and not be distracted for a long time (Duckworth et al., 2007).

Grit is most relevant to English language learners because the development of English academic language proficiency is a long-lasting practice that requires persistence (Kim and Kim, 2017; Feng and Papi, 2020) and interest (Yamashita, 2018; Chen et al., 2021). In response to the initiative proposed by Teimouri et al. (2020) to verify the domain-specify of grit, some empirical studies were conducted to explore the domain-specific versus the domain-general aspects of grit in various contexts, such as education (Schmidt et al., 2017), and L2 learning (Teimouri et al., 2020; Sudina and Plonsky, 2021). The results have consistently shown that domain-specific grit is a better predictor of achievement than domain-general grit. In the current study, a language domain-specific grit was utilized. The role of grit in foreign language learning environments has captivated scholars’ attention in recent years (Teimouri et al., 2020; Alamer, 2021). It refers to the personality traits of grit within specific foreign learning environments (Teimouri et al., 2020). Previous work on EFL learners’ grit has emphasized its positive associations with academic performance (Akos and Kretchmar, 2017; Kramer et al., 2018; Nouara and Senoussi, 2020), academic motivation, and emotions (Changlek and Palanukulwong, 2015), classroom enjoyment (Lee, 2020), scholarly persistence (Kramer et al., 2018), self-oriented learning perfectionism (Liu et al., 2021), and negative association with foreign language classroom anxiety (Li and Dewaele, 2021) and English learning burnout (Liu et al., 2021).

Most existing studies mentioned above overly focused on how EFL learners’ grit relates to academic-related outcomes while neglecting the effect of grit on other social consequences within the EFL context, such as social relationships, which are also crucial components during the learning period. In addition, grit in school learning also attracts respect and support from others (i.e., friends and teachers), and such encouragement, reflected in good interpersonal relationships, can, in turn, help overcome learning difficulties and achieve a higher sense of well-being (Leung et al., 2021). According to Lee (2020), as a motivational variable, grit encourages language learners to communicate eagerly with their peers inside the classes. Moreover, Lan (2020) demonstrated that high-grit students were more welcome than those with low-to-moderate and low grit.

Furthermore, prior work has often failed to seek the potential antecedents in the development of EFL learners’ grit, leading to limited instructions for language researchers and practitioners to promote the grit of EFL learners. School is one vital place where the impact of school capital, such as emotional support on fostering students’ positive mental features, can not be overlooked (Kothari et al., 2020). The emotional support teachers provide to their students, as a teacher-student interaction feature, is demonstrated in the way they show genuine care and concern for them, respect them, attempt to understand their feelings and points of view, and have a strong sense of dependability (Pianta and Allen, 2008; Pianta and Hamre, 2009). Such support promotes social and emotional functioning as well as learning for students (Wubbels and Brekelmans, 2005; Pianta and Hamre, 2009). The school’s emotional support is created by the interactions between teachers, students, and pupils (Jia et al., 2009). Empirical evidence has indicated that EFL learners who experience more support and care from teachers and their counterparts can utilize these positive features as effective means to surmount the challenge, which makes them more hopeful of achieving the target learning goals and motivates the development of higher levels of gritty features in their language learning (Yuan, 2022).

As mentioned above, the social relationships, including the teacher-student and peer relationships and learners’ grit, are closely intertwined within the EFL context. Most existing studies have focused on directional associations from social relationships (i.e., the teacher-student relationship, peer relationship, or both) to EFL learners’ grit (Hejazi and Sadoughi, 2022; Shen and Guo, 2022; Yuan, 2022). In contrast, limited studies were conducted to examine the effect of grit on perceived social relationships and the reciprocal associations between grit and social relationships in the EFL context. Consequently, our understanding of the nature of the relations is far from complete.

The current study addressed this issue and used a longitudinal cross-lagged design to seek the reciprocal relationships between social relationships (teacher-student and peer relationships) and grit in the EFL learning context. Unlike the existing studies (e.g., Hejazi and Sadoughi, 2022; Shen and Guo, 2022; Yuan, 2022) that only focused on one specific interpersonal relationship: the teacher-student relationship or peer relationships within the learning environment, we highlighted them synchronously because both types of interpersonal relationships exist concurrently during language learning. In addition, given that gender plays an essential effect in EFL learning (Sunderland, 1992; Ma et al., 2017; Bataineh et al., 2018; Namaziandost and Çakmak, 2020; Van Ha et al., 2021), we also investigated whether the relations between social relationships (teacher-student relationship and peer relationships) and grit within the EFL learning context differ by gender. The study could contribute to the relevant EFL learning literature by investigating the possible bidirectional associations between social relationships and grit and seeking the potential gender differences in this relation.

### The predicting effect of social relationships on grit in English as a foreign language learning

Students’ language learning can be influenced by social support from teachers and peers (Huang et al., 2010). The frequent teacher-student interactions in language classes highlight the role of teachers as an essential source of support for students during learning activities (Sadoughi and Hejazi, 2021). As a foremost component of any language teaching and learning situation, teachers play a critical role in providing instruments, appraisal, and emotional support to students (Hutchinson and Waters, 1987). Furthermore, the importance of peer support is also recognized by language learners because students study the language together and experience similar difficulties during the communication process when using the language (Huang et al., 2010).

Several possible explanations for the associations of social relationships and grit emerged in the existing literature. The first possibility is that as predictors, social relationships (the teacher-student relationship) are related to EFL learners’ subsequent grit. Most studies have addressed the importance of social relationships in fostering grit from the social-ecological perspective (Bronfenbrenner, 1979). It clarifies the underlying mechanism of the development of individuals within the social context where they live. That is, students’ context-related personality traits can be shaped within the specific learning environment. In the EFL learning context, bonding with significant others has been noted as essential in promoting grit (Shen and Guo, 2022; Yuan, 2022). Positive interpersonal interactions with teachers in the EFL context can lead to high motivation and positive emotions with more willingness to engage in language learning (Gan, 2021; Karimi and Fallah, 2021; Pishghadam et al., 2021; Sadoughi and Hejazi, 2021, 2022). Such relations can become protective factors in response to adversities and enable students to remain persistent in learning settings, promoting gritty features in EFL learning (Shen and Guo, 2022; Yuan, 2022). Numerous studies have shown that the teacher-student relationship is of great value in influencing EFL learners’ grit. For example, Yuan (2022) demonstrated that teacher stroke, referring to any action taken to acknowledge and value another person’s worth (Shirai, 2006), and teacher-student rapport, as positive teacher interpersonal behaviors, promote language learners’ grit development. What is more, findings in Hejazi and Sadoughi’s (2022) study revealed that students’ perceived teacher support was positively associated with their L2 grit, which addressed teachers’ role as an essential element in language learning to motivate students to exert considerable effort in their learning process and enhance their enthusiasm and persistence.

Positive interpersonal relationships with peers benefit students in school learning (Thapa et al., 2013). It has been shown that good peer relationships contribute to participation and engagement in school-related activities (Geng et al., 2020; Shao and Kang, 2022) and academic success (Thomas and Maree, 2022). Their peers strongly influenced adolescents’ behaviors and beliefs (Borgatti et al., 2009). Friendships and encouragement within peer groups not only offer emotional support for students but also contribute to sharing more educational resources through social connections (Chen et al., 2018). *Attachment* is a deep and enduring emotional bond that connects one person to another across time and space (Bowlby, 1969). The attachment theory (Armsden and Greenberg, 1987) demonstrated that attachment to others in social communications could promote healthy psychological characteristics, i.e., grit. Although no specific study was conducted in the EFL context to examine the effect of peer relationships on language-specific grit, the effect of general grit has been widely explored. For instance, research concerning the predicting factors of grit has shown that relatedness to friends is positively associated with the development of grit among student samples (Datu, 2017). In addition, Lan (2019) found that peer attachment is positively related to Chinese college students’ grit, indicating that positive relations with peers can promote their gritty features. Given the importance of communications with peers (guanxi, social relationships) in language learning, it is plausible to presume that peer relationships may also affect the development of grit in the EFL context.

### The predicting effect of grit on social relationships in English as a foreign language learning

The second possibility is that EFL learners’ exhibited grit would influence their subsequent interpersonal relationships with teachers and peers. Studies have found that it is crucial to examine whether EFL learners’ grit would further impact their relationships with others. The evocative effect proposed by Scarr and McCartney (1983) may interpret this association. The evocative effect refers to the individuals’ possessed features (e.g., grit) that can affect others’ attitudinal and behavioral treatment (Silinskas et al., 2015). Furthermore, the characteristics that students exhibit in the learning process (e.g., proactive, persistent vs. resistant, lazy) have been shown to facilitate or discourage interpersonal relationships (e.g., Lan, 2020). Despite the limited findings of the relationship in the EFL context, similar findings concerning the role of grit in interpersonal-related factors have emerged. For instance, studies by Lee (2020) and Lee and Lee (2020) have demonstrated that grit in foreign language learning could predict willingness to communicate, which is of utmost importance for foreign language success (Duckworth et al., 2007; Sparks et al., 2009; Baker, 2014; Keegan, 2017). Such willingness to communicate with others may be conducive to fostering good teacher-student and peer relationships (Lee, 2020).

### Reciprocal relationships between social relationships and grit in English as a foreign language learning

The third possibility is that social relationships with teachers and peers reciprocate EFL learners’ grit. The transactional theory proposed by Sameroff (2009) can be employed to explore the bidirectional relationship between social relationships and grit. This theory illustrated that students’ developmental outcomes could result from the dynamic interactions between themselves and their belonging social contexts (i.e., EFL learning context). It is plausible that social relationships (teacher-student relationship and peer relationship) in the EFL context could help shape students’ grit, which in turn, also influences students’ social relationships with teachers and peers. However, no existing studies have investigated the reciprocal linkage between social relationships (i.e., teacher-student and peer relationships) and EFL learners’ grit. Given the lack of research concerning the association between domain-specific grit and social relationships (such as teacher-student and peer relationships) in the EFL context, it is, therefore, essential to conducting in-depth research on this relationship, which is beneficial for pedagogic practice intervention.

### Gender differences in social relationships and grit in English as a foreign language learning

Gender plays a crucial role in learners’ foreign language learning (Sunderland, 1992; Bataineh et al., 2018; Namaziandost and Çakmak, 2020; Van Ha et al., 2021), social relationships (Eranil and Özcan, 2019), and the formation of grit (Christensen and Knezek, 2014; Fernández Martín et al., 2020). Studies have suggested considerable differences between boys’ and girls’ gritty traits in English. Richardson and Woodley’s (2003) study noted that females tended to obtain higher academic achievement than males because they worked harder and persisted more than males. What is more, Feng et al.’s (2013) study supported this perspective indicating that girls were more motivated to learn English and willing to demonstrate good learning behaviors. Previous studies have suggested that teachers might treat girls with more leniency than boys. In other words, to improve boys’ performance, English language teachers would adopt more discipline and control over boys than girls; thus, boys may perceive a lower positive teacher-student relationship from their English language teachers than girls (Jones and Wheatley, 1990). There has been consistent evidence of gender differences in how people perceive social relationships. For instance, Eranil and Özcan (2019) found that high school female students reported statistically significantly higher scores than male students concerning their social relations with peers. Previous studies on the development of grit, perceived teacher-student, and peer relationships would treat gender as a demographic variable to test gender difference by comparing the mean differences (Christensen and Knezek, 2014; Eranil and Özcan, 2019; Fernández Martín et al., 2020) or treat it as a control variable when testing the associations among variables (e.g., Ma et al., 2017). It is significant to make a meaningful multi-group comparison across gender to determine whether the associations between social relationships and grit differ by gender. The results of such comparison in the structural model could help educators better understand the cross-gender applicability of the cross-lagged analytic model.

### The Chinese English as a foreign language context

English as a foreign language learners in China account for a large proportion of English learners in the world because English course is one of the compulsory courses in the compulsory education stage for most Chinese students, and the English language proficiency test is also brought into the high school entrance examination and college entrance examination (Ministry of Education of the People’s Republic of China [MOE], 2001). Therefore, the study of the Chinese EFL sample has great relevance to other EFL learners. However, some Western researchers have been concerned that the language learning process for Chinese students is dependent and passive, which may reflect a lack of grit in language learning (Hiver et al., 2021). In other words, students who lack gritty traits are theoretically presumed to be less persistent in learning English and tend to give up when meeting setbacks. Some researchers have asserted that this phenomenon is mainly due to the coercive and repressive teachers (Jiang et al., 2021) during classroom learning and highly competitive peer relationship (Ellicott, 2013; Li, 2017; Chen and Hu, 2022). Given the perspectives mentioned above, it is of importance for Chinese educators to investigate the possible dynamic association between the social relationship in the English learning environment and the development of Chinese EFL learners’ grit. The findings of our study may contribute to solving this issue to some extent.

### The current study

Most of the existing studies have employed cross-sectional research designs to explore the unidirectional associations between social relationships and grit in EFL learning: concerning social relationships as predictors to influence students’ grit (Eskreis-Winkler et al., 2014; Datu, 2017; Jin et al., 2019; Lan, 2019; Hejazi and Sadoughi, 2022; Yuan, 2022) or concerning students’ grit to predict social relationship related variables (Lee, 2020). However, such associations might be dynamic and bidirectional. Longitudinal studies should be an appropriate approach to fully understand how social relationships (teacher-student and peer relationships) and EFL learners’ grit influence each other over time. Therefore, the current study examined the reciprocal associations between social relationships and grit within the EFL context among Chinese high school students. We simultaneously addressed the effects of the teacher-student and peer relationships, which could depict a comprehensive picture of social relationships in the EFL learning context. We also employed a three-wave cross-lagged analytic approach, given that the cross-lagged analytical approach provided a more rigorous way to check the constructs’ stability across time points and the reciprocal linkage between constructs.

Guided by the above findings, we proposed the study’s research hypotheses (RHs). After controlling for auto regressor effects of each construct,

RH1. Time 1 and Time 2 social relationships (teacher-student and peer relationships) would be separately associated with Time 2 and Time 3 grit in EFL learning.

RH2. Time 1 and Time 2 grit would be separately associated with Time 2 and Time 3 social relationships (teacher-student and peer relationships) in EFL learning.

RH3. Such relations between social relationships (the teacher-student relationship and peer relationships) and grit in EFL learning would differ regarding students’ gender.

## Materials and methods

### Participants

We recruited students from four public high schools in a northeast city of China. The students (*n* = 2,719) in the affiliated schools participated in the study voluntarily. We eliminated 284 students (9.41%) who did not take the test all three times. Among the remaining 2,435 students, 1,116 (45.8%) were boys, and 1,319 (54.2%) were girls. The mean age of the students was 16.40 years old (SD = 0.84). All the students speak Chinese as their first language and are of Han nationality.

### Measures

Results of the reliabilities (internal consistency) and the psychometric properties indicated that the following scales were reliable and valid (see Table 1) at each time point.

**TABLE 1:** The reliability coefficients and the psychometric properties of the tested scales.

	*n*	α	CFI	TLI	SRMR	RMSEA (90%CI)
**Time 1**						
Teacher-student relationship	5	0.91	0.99	0.98	0.01	0.074 (0.060–0.088)
Peer relationship	10	0.87	0.97	0.95	0.03	0.070 (0.064–0.076)
Grit	9	0.78	0.98	0.97	0.03	0.057 (0.050–0.064)
**Time 2**						
Teacher-student relationship	5	0.95	0.99	0.98	0.01	0.076 (0.069–0.091)
Peer relationship	10	0.88	0.98	0.98	0.03	0.061 (0.055–0.067)
Grit	9	0.75	0.98	0.98	0.03	0.059 (0.052–0.066)
**Time 3**						
Teacher-student relationship	5	0.97	0.99	0.99	0.01	0.073 (0.059–0.087)
Peer relationship	10	0.89	0.98	0.97	0.02	0.070 (0.065–0.076)
Grit	9	0.76	0.98	0.98	0.02	0.057 (0.050–0.065)

#### Teacher-student relationship

Students’ perceived teacher-student relationship was measured with the Chinese-version questionnaire in the Programme for International Student Assessment ([PISA], Organization of Economic Co-Operation and Development [OECD], 2013). Five items were to examine the interaction between students and their English language teachers. A sample item included “I can get along well with my English teachers.” Students responded to the scale using a 5-point Likert-type scale ranging from 1 (*strongly disagree*) to 5 (*strongly agree*). Higher scores represented better relationships with English language teachers during English class. The test-retest reliability coefficients ranged from 0.29 to 0.42, indicating considerable changes in students’ perceived degrees of the teacher-student relationship across three-time points.

#### Peer relationship

To measure students’ perceptions of the closeness between peers during English learning, we used the ten-item Peer Closeness Scale. It was developed by the National Children’s Study of China (NCSC) in China and has been used many times in large-scale assessments in mainland China. We chose this scale to measure peer closeness due to its satisfactory reliabilities and psychometric properties among various regional and school-stage samples in mainland China (Dong and Lin, 2011; Liu and Liu, 2021). Students completed ten items using a 4-point Likert scale ranging from 1 (*strongly disagree*) to 4 (*strongly agree*) to reflect the degrees of peer interactions in English learning. A sample item included, “I am happy when I am with my classmates.” The test-retest reliability coefficients ranged from 0.36 to 0.52, indicating substantial changes in students’ perceived levels of peer relationship across three-time points.

#### Grit

The measure of students’ grit in the EFL context was deployed by Teimouri et al. (2020). It consisted of 9 items with two dimensions: consistency of interest (e.g., My interests in learning English change from year to year, 4 items) and perseverance of effort (e.g., I am a diligent English language learner, 5 items). It is a newly developed language domain-specific scale regarding the concept of grit, which could better reflect students’ language-specific gritty features than using the general Grit Scale (developed by Duckworth et al., 2007). Students responded to 9 items on a 4-point Likert scale ranging from 1 (*not like me at all*) to 4 (*very much like me*). Items in the perseverance of effort dimension were regular-scored, and those in the consistency of interest dimension were reversed-scored. A higher score represented that students possessed a higher level of gritty traits in learning English.

### Data collection procedures

We first obtained approval from the target school principals and the English course teachers. Upon support from principals and teachers, students’ parents were firstly distributed a consent form to determine their willingness to allow their child to participate in the study. A separate consent form was then sent to students to specify whether they voluntarily answered the online questionnaire; once they agreed, they completed the questionnaire during English class to minimize the external noise. We administered the survey three times, and students were asked to respond to the same measures in each survey. The first survey was administered in November 2020, the second in May 2021, and the third in November 2021. The interval between every two surveys was approximately 6 months, and the investigators did not use any intervention during these dates.

### Data analytic procedures

We first calculated preliminary analysis, including the descriptive statistics, the bivariate correlation, and internal consistency reliability (Cronbach’s alphas) using SPSS 24.0. Pearson correlation coefficients around 0.10 indicated a small effect size, around 0.03 indicated a moderate effect size, and higher than 0.50 reflected a strong effect size (Cohen, 1992). Second, we conducted cross-sectional Confirmatory factor analysis (CFAs) each time. We employed multiple goodness of fit indices, entailing comparative fit index (CFI), Tucker-Lewis Index (TLI), standardized root mean square residual (SRMR), and root mean square of error of Approximation (RMSEA), to evaluate the CFA models. We considered the values of CFI and TLI greater than 0.90, the value of SRMR less than 0.06, and the value of RMSEA less than 0.08, as the indicators of an acceptable fit to the data (Hu and Bentler, 1999; Hooper et al., 2008; Hair et al., 2010). Third, we conducted a multiple-group confirmatory factor analysis (MG-CFA) across three-time points *via* a maximum likelihood estimation approach to check whether the constructs held the same meaning over time in AMOS 24.0. More specifically, we tested configural invariance (invariance of the overall factor structure), metric invariance (invariance of factor loadings), and scalar invariance (invariance of item intercepts) of the constructs across three-time points. We employed two recommended criteria shown in previous studies to evaluate the longitudinal factorial invariance of the measurement models (e.g., Datu and King, 2018). The changes in CFI value less than 0.01 and in TLI less than 0.05 represented longitudinal measurement invariance (Little, 1997; Cheung and Rensvold, 2002).

Fourth, we tested a longitudinal CFA to determine whether the measurement model across times is valid. The model comprised all the constructs at three-time points. We correlated the residual errors of each latent construct at three-time points, which followed the notion of Marsh and Yeung (1998), indicating that such an approach could gain more precise path coefficients in a structural model. Fifth, we conducted a three-wave cross-lagged path analysis using a maximum likelihood estimation approach to test the associations between the teacher-student relationship, peer relationship, and students’ grit in the Chinese EFL context. We also controlled the auto regressor effects of variables in the model. Concerning the effect sizes of the cross-lagged coefficients, we followed the effect size guidelines that were popular in educational research (Keith, 2015), where the standardized coefficients smaller than 0.05 reflected meaningless effects; the standardized coefficients between 0.05 and 0.10 reflected small effects; the standardized coefficients between 0.10 and 0.25 reflected moderate effects, and the standardized coefficients higher than 0.25 reflected large effects. Finally, we performed an MG-CFA to check whether the cross-lagged path model between social relationships and grit differ across gender. We examined the invariance of the structural model with structural weights, structural intercepts, structural means, and structural variances and covariances (Byrne, 2004). The evaluation criteria are the same as above.

## Results

### Preliminary analysis

The result of the preliminary analysis presents in Table 2. The teacher-student relationship, peer relationship and EFL learners’ grit were significantly and positively correlated in each time (Time 1: *r* ranged from 0.38 to 0.54, *p*s < 0.001; Time 2: *r* ranged from 0.39 to 0.58, *p*s < 0.001; Time 3: *r* ranged from 0.37 to 0.63, *p*s < 0.001).

**TABLE 2:** Descriptive statistics and pearson correlation coefficients among variables.

	*M*	SD	1	2
**Time 1**				
1. Teacher-student relationship	4.13	0.84	–	
2. Peer relationship	3.46	0.53	0.41*	–
3. Grit	3.18	0.56	0.41*	0.54*
**Time 2**				
1. Teacher-student relationship	4.09	0.89	–	
2. Peer relationship	3.34	0.59	0.44*	–
3. Grit	3.12	0.57	0.43*	0.58*
**Time 3**				
1. Teacher-student relationship	4.17	0.91	–	
2. Peer relationship	3.32	0.61	0.38*	–
3. Grit	3.11	0.63	0.38*	0.63**

*p < 0.001.

### Cross-sectional measurement models

Table 3 presents the results of cross-sectional CFAs concerning the measurement models to be tested at each time point. Each measurement model is composed of three latent factors: one for the teacher-student relationship, one for peer relationships, and one for grit. The CFA model results indicated that the measurement model was valid at all three-time points. All the indicators were shown to be significantly loaded on the corresponding latent factor (*p*s < 0.001).

**TABLE 3:** Cross-sectional CFAs for Time 1, Time 2, and Time 3.

	χ*^2^*	*df*	CFI	TLI	SRMR	RMSEA (90%CI)
Time 1 model	1944.12*	236	0.95	0.94	0.05	0.055 (0.052–0.057)
Time 2 model	2193.59*	236	0.96	0.95	0.05	0.058 (0.056–0.061)
Time 3 model	2934.28*	236	0.96	0.96	0.06	0.061 (0.059–0.064)

*p < 0.001.

### Multi-group invariance test for the measurement model across time points

Results of MG-CFA regarding the measurement model across three-time points are presented in Table 4. The configural invariance model fitted the data well, with χ*^2^*(708) = 6531.99, CFI = 0.96, TLI = 0.95, SRMR = 0.05, RMSEA = 0.034 (90% CI:0.033–0.034), indicating that the overall factor structure remained stable across three time points. Then, metric invariance model was also satisfied across three time points, with χ*^2^*(750) = 6834.34, CFI = 0.95, TLI = 0.95, SRMR = 0.06, RMSEA = 0.033 (90% CI:0.033–0.034), Δ*CFI* (M_metric_ - M_configural_) = 0.002, Δ*TLI* (M_metric_ - M_configural_) < 0.001, representing the invariance of factor loadings was held. Next, the scalar invariance model also fitted the data well, with χ*^2^*(798) = 7794.31, CFI = 0.95, TLI = 0.95, SRMR = 0.06, RMSEA = 0.035 (90% CI:0.034–0.035), Δ*CFI* (M_scalar_-M_metric_) = 0.007, Δ*TLI* (M_scalar_-M_metric_) = −0.004, meaning that the item intercepts were invariant, and the respondents similarly interpreted items across times. The relatively small changes of CFI and TLI abovementioned demonstrated that the measurement model was invariant across the three-time points.

**TABLE 4:** Testing for factorial invariance across three time points.

	χ*^2^*	*df*	CFI	Δ *CFI*	TLI	Δ *TLI*	SRMR	RMSEA (90%CI)
Configural invariance model	6531.99*	708	0.96		0.95		0.05	0.034 (0.033–0.034)
Metric invariance model	6834.34*	750	0.95	−0.002	0.95		0.06	0.033 (0.033–0.034)
Scalar invariance model	7794.31*	798	0.95	−0.007	0.95	+0.004	0.06	0.035 (0.034–0.035)

*p < 0.001.

### Longitudinal measurement model

In the longitudinal CFA model, each of the three latent constructs separately represented the teacher-student relationship, peer relationship, and EFL learners’ grit at time 1, time 2, and time 3. The final model contained nine latent factors in the three-time points. The model showed adequate fit indices, with χ^2^ (2,427) = 11987.38, *p* < 0.001, CFI = 0.93, TLI = 0.93, SRMR = 0.03, RMSEA = 0.040 [90% CI:0.040–0.041]. All the indicators were significantly loaded to the corresponding latent factor at *p* < 0.001. Therefore, the results abovementioned indicated that all the examined constructs showed similar meanings and temporal stability across three-time points.

### Cross-lagged panel model

**FIGURE 1:**
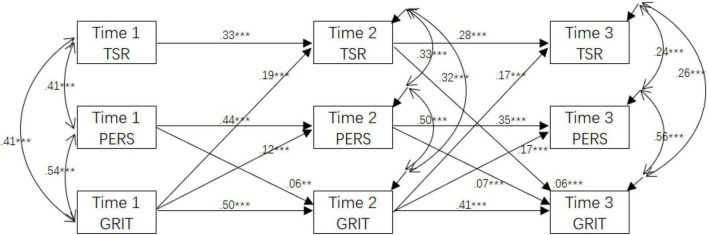
Cross-lagged path analysis model on the relationships between social relationship and grit in the English as a foreign language (EFL) context. TSR, teacher-student relationship; PERS, peer relationship. **p < 0.01, and ***p < 0.001.

The cross-lagged model fitted the data well, with χ^2^ (13) = 227.56, *p* < 0.001, CFI = 0.97, TLI = 0.92, SRMR = 0.05, RMSEA = 0.080 [90% CI:0.073–0.092]. Most of the paths in the model were statistically significant; nevertheless, we found that the teacher-student relationship in Time 1 did not significantly associate with EFL learners’ grit in Time 2 (β = 0.01, *p* = 0.388). Therefore, we tested an alternative model without the non-significant path (from Time 1 teacher-student relationship to Time 2 grit). The alternative model also revealed good model fit to the data, χ^2^ (14) = 228.31, *p* < 0.001, CFI = 0.97, TLI = 0.93, SRMR = 0.05, RMSEA = 0.079 [90% CI:0.070–0.089]. Due to the non-significant chi-square test between the hypothesized and alternative models (Δχ*^2^* = 0.75, *df* = 1, *p* > 0.05), we decided to employ the alternative model as our final model due to the more parsimony of that model than the initial one (see Figure 1).

Results of the alternative model showed that all the constructs at Time 1 could significantly predict their Time 2 counterparts, and the constructs at Time 2 could significantly predict their Time 3 counterparts. At Time 1, only peer relationships positively predicted Time 2 grit in EFL learning (β = 0.06, *p* < 0.01, small effect size). However, in line with our expectations, both teacher-student relationship (β = 0.06, *p* < 0.01, small effect size) and peer relationships (β = 0.07, *p* < 0.01, small effect size) at Time 2 could positively predict Time 3 grit in EFL learning. RH1 was partially supported concerning the association between social relationships (teacher-student and peer relationships) and students’ subsequent grit in EFL learning.

Results showed that Time 1 grit in EFL learning could positively predict Time 2 teacher-student relationship (β = 0.19, *p* < 0.001, moderate effect size) and peer relationships (β = 0.12, *p* < 0.001, moderate effect size); Time 2 grit in EFL learning could positively predict Time 3 teacher-student relationship and peer relationships (βs = 0.17, *p*s < 0.001, moderate effect sizes). Findings supported RH2, demonstrating that students’ grit in EFL learning is associated with their subsequent social relationships (teacher-student and peer relationships).

In sum, we conclude that the hypothesis of the reciprocal links between social relationships and grit in EFL learning was partially supported: peer relationships showed reciprocal effects with grit in both two-time lags. The teacher-student relationship is influenced by grit in the first-time lags and then showed reciprocal effects with grit in the second-time lags. The effect is slightly more substantial regarding the prediction of grit on the social relationship than the reversed predictive relationship.

### Multi-group invariance test for the structural model across gender

Results of MG-CFA regarding the structural model across gender are presented in Table 5. All the invariance models fitted the data well, and the model comparisons demonstrated the relatively small changes in values of CFI and TLI. Findings indicated that the relations between social relationships (the teacher-student relationship and peer relationships) and grit in EFL learning remained stable across gender.

**TABLE 5:** Testing for structural invariance across gender.

	χ*^2^*	*df*	CFI	Δ *CFI*	TLI	Δ *TLI*	SRMR	RMSEA (90%CI)
Unstrained model	239.552*	28	0.97		0.95		0.05	0.056 (0.049–0.062)
Structural weights invariance model	269.824*	41	0.97	−0.002	0.95	+0.001	0.05	0.048 (0.043–0.053)
Structural intercepts invariance model	321.998*	47	0.96	−0.006	0.95	−0.003	0.05	0.049 (0.044–0.054)
Structural means invariance model	355.964*	50	0.96	−0.004	0.94	−0.003	0.05	0.050 (0.045–0.055)
Structural variances and covariances invariance model	391.337*	56	0.96	−0.004	0.94	+0.002	0.06	0.050 (0.045–0.054)

*p < 0.001.

## Discussion

The current study used three-wave data to explore the bidirectional relationship between social relationships (the teacher-student relationship and peer relationship) and grit among Chinese EFL students. The cross-lagged model indicated that peer relationships and EFL students’ grit were positively and reciprocally related across time points. In contrast, the teacher-student relationship and EFL students’ grit only showed positive reciprocal associations from Time 2 to Time 3. During the first time interval, the Time 1 teacher-student relationship was not significantly associated with EFL students’ Time 2 grit, yet students’ Time 1 grit would positively predict their perceptions of the teacher-student relationship at Time 2. The findings provided evidence to understand better the dynamic associations between social relationships (the teacher-student and peer relationships) and students’ grit within the Chinese EFL context.

Our results partially supported the RH1 showing that EFL students perceived peer relationships in Time 1 and Time 2 positively and significantly related to Time 2 and Time 3 grit. However, only Time 2 teacher-student relationship was positively and significantly associated with Time 3 subsequent grit. The findings echoed previous studies (Hejazi and Sadoughi, 2022; Shen and Guo, 2022; Yuan, 2022). When students have positive interpersonal relationships in EFL learning, they may involve in a friendly learning climate and benefit from other’s support and care, which not only helps them relieve anxiety and pressure in learning English (Piechurska-Kuciel, 2011; Hung, 2020) but also inspires them to stick to their learning goals (Harter, 1996). It should be noted that the teacher-student relationship at Time 1 was not significantly related to EFL students’ grit at Time 2. The possible reason is that our study started 2 months after they entered high school; students and English language teachers are not yet familiar with each other, so the prediction effect is negligible. As the time that teachers interacted with students increased, the influence of English language teachers on EFL students’ outcomes gradually increased and started to benefit students’ subsequent grit development.

Conversely, peer relationships at Time 1 could significantly predict grit at Time 2. This significant association may be because there was already a degree of familiarity between the peers during the study. One possibility was that before the start of this study, the high school students had already completed military training (a required course in high school). The students got along with each other in the military training process and gradually became familiar with each other. Another possibility was that with only four high schools in the tested district, which is limited in size, many students might have been familiar with each other before entering the high schools. Unfortunately, such information was not available in our background information, so it is just a possibility worth explaining the findings.

Our results fully supported the RH2, showing EFL students’ grit in Time 1 and Time 2 related to Time 2 and Time 3 social relationships (teacher-student and peer relationships). It was in line with previous findings demonstrating that grit could predict willingness to communicate with others (e.g., Lan, 2020; Lee, 2020; Lee and Lee, 2020). Our findings verified the notion of the evocative effect, indicating that students’ developmental outcomes (i.e., grit) would impact others’ (e.g., teachers and friends) subsequent attitudes and behaviors toward them. When students exhibit more positive features in learning, such performance may be satisfied by teachers, and in turn, teachers would treat them in a more friendly manner. Likewise, an old Chinese saying goes, “Jin zhu zhe chi, jin mo zhe hei”, a good environment can improve a person; however, a bad environment can worsen a person. Students who possess more grit are good role models among peers. They are also more likely to encourage and drive others to persevere in learning together, leading to closer friendships (Lan, 2020).

In terms of gender difference, the current study found no significant gender difference in the cross-lagged associations between social relationships and EFL students’ grit, contrasting RH3, which is consistent with previous studies (Duckworth and Quinn, 2009; Vainio and Daukantaitë, 2016; Lee, 2020). The findings indicated that the strength in such associations for males and females was identical. Although male and female students may perceive interpersonal relationships differently and possess grits diversely, relationship intensity is consistent, which indirectly indicates that interpersonal relationships and grits are correlated to the same degree, regardless of gender.

### Limitation and further direction

Several study limitations merit attention. First, the teacher-student and peer relationships we examined were from data collected from a self-reported questionnaire. It might not accurately reflect the relationships they indeed received from teachers of English and peers, leading to a subjective conclusion due to social desirability bias. Further research can include other ratings (e.g., self- vs. teacher- and self- vs. peer- ratings) on these social relationships into the research design to determine the similarities and differences concerning these relations across various raters. Second, the current study was an overpowering correlational research design; thus, we could not draw causal conclusions from the tested factors. Future research might apply an experimental research design to address this concern.

Third, the student samples we recruited in the study only came from one city in China; this was not representative of all Chinese EFL students. Therefore, the generalization of the results will be limited to some extent. In future work, students can be recruited from different types of schools across the country or even from other countries with various cultural backgrounds to ensure the sample’s diversity. Fourth, measures that focus on peer relationships in English learning are limited, and this study is one of the first to adopt the general measure of peer relationships in a foreign language learning context. Therefore, we call for researchers to adopt a more valid context-specific instrument of peer relationships that may capture more specific EFL features to replicate our study.

## Conclusion

The teacher-student and peer relationships are crucial social relationships that facilitate students’ personality development. The current study employed a cross-lagged panel model to better understand the underlying mechanisms in EFL students’ personality developmental process to facilitate specific interventions for EFL learning. Our findings suggested a symmetrically positive reciprocal relationship between peer relationships and EFL learners’ grit. Such symmetrically positive reciprocal relationships also existed between the teacher-student relationship and grit when the English language teacher and students got familiar with each other, but an asymmetrical pattern that only grit positively predicted the teacher-student relationship when they were unfamiliar with each other.

### Practical implications

The findings suggest the vital role of social relationships in positive personality development within the EFL context. Students exhibited performance in the English learning process also has an evocative effect leading to different social relationships with others. English language teachers should establish friendly interactions providing more care and support to students. This support can range from emotional encouragement to pedagogical support to promote persistence and willingness to communicate in English. They can also employ more cooperative learning tasks to promote the close relationship between students. Positive peer interactions can stimulate interest in using and learning English.

## Data availability statement

The raw data supporting the conclusions of this article will be made available by the authors, without undue reservation.

## Ethics statement

The studies involving human participants were reviewed and approved by the University of Macau Research Services and Knowledge Transfer Office. Written informed consent to participate in this study was provided by the participants’ legal guardian/next of kin.

## Author contributions

TC significantly contributed to the conception of the study, designed and administered the survey, performed the data analyses, and wrote the manuscript. YY contacted participants, performed the analysis with constructive discussions, and contributed to analysis and manuscript preparation. Both authors contributed to the article and approved the submitted version.
